# Delayed Laboratory Response to COVID-19 Caused by Molecular Diagnostic Contamination

**DOI:** 10.3201/eid2608.201843

**Published:** 2020-08

**Authors:** Ramona Mögling, Adam Meijer, Natasa Berginc, Sylvia Bruisten, Remi Charrel, Bruno Coutard, Isabella Eckerle, Vincent Enouf, Olav Hungnes, Gülay Korukluoglu, Thanos Kossyvakis, Andreas Mentis, Richard Molenkamp, Shaman Muradrasoli, Anna Papa, Fiona Pigny, Laurence Thirion, Sylvie van der Werf, Chantal Reusken

**Affiliations:** National Institute for Public Health and the Environment, Bilthoven, the Netherlands (R. Mögling, A. Meijer, C. Reusken);; Laboratory for Public Health Virology, Ljubljana, Slovenia (N. Berginc);; Public Health Service of Amsterdam, Amsterdam, the Netherlands (S. Bruisten);; Aix Marseille Université, Marseille, France (R. Charrel, B. Coutard, L. Thirion);; University Hospital Geneva, Geneva, Switzerland (I. Eckerle, F. Pigny);; Institut Pasteur, Paris, France (V. Enouf, S. van der Werf);; Norwegian Institute of Public Health, Oslo, Norway (O. Hungnes);; Public Health General Directorate of Turkey, Ankara, Turkey (G. Korukluoglu);; Hellenic Pasteur Institute, Athens, Greece (T. Kossyvakis, A. Mentis);; Erasmus Medical Center, Rotterdam, the Netherlands (R. Molenkamp, C. Reusken);; Public Health Agency of Sweden, Slona, Sweden (S. Muradrasoli);; Aristotle University of Thessaloniki, Thessaloniki, Greece (A. Papa)

**Keywords:** delayed laboratory response, severe acute respiratory syndrome coronavirus 2, SARS-CoV-2, coronaviruses, viruses, coronavirus disease, COVID-19, respiratory infections, contamination, PCR, primers, probes, molecular diagnostics, commercial nucleotide production, zoonoses, Europe

## Abstract

The emergence of severe acute respiratory syndrome coronavirus 2 (SARS-CoV-2) created an exceptional situation in which numerous laboratories in Europe simultaneously implemented SARS-CoV-2 diagnostics. These laboratories reported in February 2020 that commercial primer and probe batches for SARS-CoV-2 detection were contaminated with synthetic control material, causing delays of regional testing roll-out in various countries.

Timely and reliable laboratory diagnosis is crucial for clinical care and to inform public health responses in the ongoing severe acute respiratory syndrome coronavirus 2 (SARS-CoV-2) pandemic (*1*). The laboratory response in Europe to emergence of SARS-CoV-2 appeared rapid at the country level; 38 laboratories in 24 European Union/European Economic Area countries had molecular testing already available by January 29, 2020, and an expected complete coverage of all European Union/European Economic Area countries by mid-February (*1*).

The first protocol for molecular detection, with a focus on envelope (E) and RNA-dependent RNA polymerase gene targets, was available on January 13, 2020 (*2*,*3*), and shared rapidly. Toward the end of January 2020, reports from laboratories in Europe indicated that commercial, custom-made primer and probe batches for SARS-CoV-2 detection might be contaminated with synthetic control material for the E gene target. This observation was disclosed within the expert laboratory network for Emerging Viral Diseases–LabNet (*4*) on February 5, 2020, and resulted in an alert and advice to perform a second target confirmation by the European Centre for Disease Prevention and Control (ECDC) on its website (*5*). A call for more detailed information was send out to assess the extent of the situation.

Ten laboratories from 8 countries in Europe reported PCR template contamination in commercially ordered primer and probe batches, which led to SARS-CoV-2 reverse transcription PCR (RT-PCR) signals in their no-template controls, and provided detailed information. Five additional laboratories (including addition of a ninth affected country) indicated that they received contaminated material but did not provide details.

Materials were ordered during January 13–February 28 from 8 companies offering custom nucleic acid synthesis. Delivery of contaminated oligonucleotides was reported during January 22–February 28 for 6 companies, including those that initially delivered contamination-free oligonucleotides until January 21 (Figure). The contamination issues concerned primer and probe batches for the E and the RNA-dependent RNA polymerase gene targets, as well as batches for nonrelated targets received on the same day. Others reported sporadic contamination. The extent of contamination varied strongly; reported cycle threshold values ranged from 23 to 39. The laboratories systematically excluded other, own laboratory-related, potential sources of contamination. None of the 10 laboratories ordered long synthetic DNA polymers.

**Figure:**
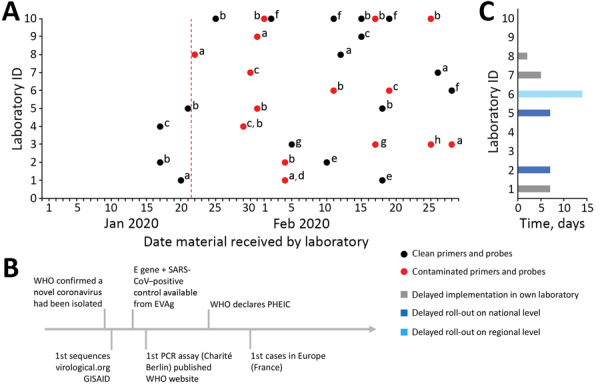
Timeline and extent of product and molecular diagnostic contamination issues in 10 laboratories in Europe during delayed laboratory response to COVID-19. A) Contamination status of commercially ordered primers and probes for molecular detection of SARS-CoV-2 based on Corman et al. (*2*). Red vertical dotted line indicates starting date of laboratories in Europe receiving contaminated commercial primers and probes. The letters a–h are unique identifiers for the 8 companies that produced the materials. B) Timeline of simultaneous hallmark events in the SARS-CoV-2 outbreak. C) Delay of implementation of SARS-CoV-2 diagnostic test in laboratories and delay of national or regional roll-out schemes per laboratory. Laboratories that indicated no delay had access to noncontaminated material from previous orders or cooperated with another laboratory. COVID-19, coronavirus disease; E, envelope; EVAg, European Virus Archive Global; GISAID, Global Initiative on Sharing All Influenza Data (http://gisaid.org); ID, identification; PHEIC, public health emergency of international concern; SARS-CoV, severe acute respiratory syndrome coronavirus; SARS-CoV-2, severe acute respiratory syndrome coronavirus 2; WHO, World Health Organization.

Six laboratories indicated a delayed implementation of SARS-CoV-2 diagnostics. Three were central laboratories responsible for roll-out of diagnostic capability to regional and hospital laboratories within their country, which was therefore delayed by 7–14 days. Three laboratories indicated a delay in molecular test implementation of 2–7 days in their own facilities (Figure, panel C). One laboratory described a delay in final negative result reporting for 1 suspected patient during a tense period in which the country did not have any cases.

The companies involved were informed. Some offered new batches free of charge, started to screen their products postproduction, or stopped production of long oligonucleotides. Others did not respond or denied that a problem existed. One company decontaminated its production facility.

The emergence of SARS-CoV-2 created an exceptional situation that demanded a rapid implementation of RT-PCRs. We hypothesize that the combined simultaneous and huge demand across Europe for primers, probes, and controls, related to the protocol of Corman et al. (*2*), might have led to production of primers and probes contaminated with synthetic controls. Initial limited access to positive controls (*1*) might have led to orders of long synthetic DNA polymers spanning SARS-CoV-2 RT-PCR target genes. In combination with extensive and simultaneous ordering of associated primers and probes, this ordering resulted in synthesis on the same production line within a short time span or in close proximity within some companies.

Companies that produce custom synthetic nucleotides need to be aware of these potential problems that might only appear in extreme situations, such as the massive laboratory response to SARS-CoV-2 at the end of January/beginning of February 2020 in Europe that was uniform and based on few available protocols (*2*). In normal circumstances, the common practice of synthesis of primers, probes, and long nucleic acids would not necessarily pose a major problem because different nucleic acids are randomly ordered and produced. However, in an emergency response scenario as described here, this common practice had consequences for an efficient laboratory and public health response.

Comparison of ordered nucleic acids against sequence databases might inform the synthesis set-up at companies. This comparison could be combined with the already existing protocol for nucleic acid–synthesizing companies regarding synthesis of high-risk pathogens (*6*). Other measures might include separate production facilities for long and short nucleic acids. The necessity for this change was highlighted by a 16th laboratory that failed to order their primers and probes through explicit routing of a company to avoid contamination with popular PCR targets. E gene–contaminated primers and probes were received at the end of March 2020.

This report provides a warning to manufacturers of oligonucleotides and diagnostic laboratories alike to remain vigilant for contamination issues in popular RT-PCR reagents. Vigilance will help avoid delays in crucial laboratory responses now and in future outbreak events.
